# Development of a One-Pot MIRA-CRISPR/Cas12b Fluorescence Biosensing Platform for Rapid Detection of *Brucella canis*

**DOI:** 10.3390/bios16100578

**Published:** 2026-10-15

**Authors:** Suxin Huo, Yinchu Zhu, Congyun Ji, Fu Fan, Ruiping Lin, Weicheng Ye, Yuan Fu, Jionggang Hua, Gaole Feng, Liu Chen, Tao Yun, Cun Zhang, Zhuanli Wu, Xing Xu, Yue Wu, Zheng Ni

**Affiliations:** 1 State Key Laboratory for Quality and Safety of Agro-Products, Key Laboratory of Animal and Poultry Breeding of Zhejiang Province, Key Laboratory of Livestock and Poultry Resources (Poultry) Evaluation and Utilization, Institute of Animal Husbandry and Veterinary Science, Zhejiang Academy of Agricultural Sciences, Hangzhou 310021, China; huosuxin831@163.com (S.H.); zhuyinchu@zaas.ac.cn (Y.Z.);; 2 Pinghu Animal Husbandry and Veterinary Station, Pinghu 314200, China

**Keywords:** *Brucella canis*, biosensor, MIRA, CRISPR/Cas12b, clinic diagnostic

## Abstract

Brucellosis is an important zoonotic disease worldwide, and *Brucella canis* (*B. canis*) is an emerging concern in companion animal health. Rapid and reliable detection of *B. canis* in dogs is essential for disease control. Herein, we developed a one-pot MIRA CRISPR/Cas12b fluorescence biosensing platform targeting the conserved *omp31* gene. The platform integrates MIRA mediated isothermal amplification with CRISPR/Cas12b-based target recognition and collateral cleavage. After optimization, the assay achieved a detection limit of 5 copies/reaction within 20 min, comparable to qPCR. No cross reactivity was observed with seven common canine pathogens. Among 128 clinical blood samples, the biosensor showed complete agreement with qPCR, achieving 100% sensitivity and 100% specificity. These findings demonstrate that the biosensor provides a rapid, sensitive, and specific approach for *B. canis* detection, with potential for decentralized veterinary testing through its endpoint visual fluorescence readout and applications in One Health surveillance.

## 1. Introduction

Pet dogs are playing an increasingly important role in modern society, with the canine population in urban China reaching 53.43 million in 2025 according to the 2026 China Pet Industry White Paper. This rapid expansion of companion animal populations has heightened concerns regarding canine infectious diseases and their potential zoonotic implications [1]. Among these diseases, *brucellosis*—a globally distributed bacterial disease—is a serious threat to animal and human health, listed as a Class II animal disease by the Ministry of Agriculture and Rural Affairs of China and notifiable by the World Organisation for Animal Health (WOAH) [2].

Canine brucellosis, primarily caused by *B. canis*, is associated with reproductive and systemic disorders in dogs, including abortion, infertility, orchitis, epididymitis, discospondylitis, endocarditis, and uveitis [3,4,5]. While rarely fatal, the infection is often chronic and difficult to eradicate, resulting in long-term health consequences and substantial economic losses in breeding operations. Importantly, infected dogs may serve as a source of zoonotic transmission, posing potential risks to veterinarians, kennel workers, dog owners, children, and immunocompromised individuals through exposure to infected animals and contaminated reproductive or other bodily secretions [6,7,8,9]. Early infection is frequently asymptomatic or associated with nonspecific clinical manifestations, which can facilitate unnoticed transmission and complicate timely diagnosis. Therefore, rapid, sensitive, and reliable detection of *B. canis* is essential for early identification, clinical management, and effective surveillance and control of canine brucellosis.

Bacterial isolation remains the gold standard for *Brucella* diagnosis, but culture-based detection is technically demanding and time-consuming, limiting its suitability for rapid screening and on-site testing [10,11]. Serological methods provide alternative approaches but also have inherent limitations. The microplate serum agglutination test (mSAT) is suitable for large-scale screening but may suffer from limited specificity, whereas the complement fixation test (CFT), although offering improved specificity, is labor-intensive and less amenable to field applications. Immunofluorescence antibody testing (IFAT) can provide high diagnostic sensitivity and specificity but requires specialized equipment and trained personnel, restricting its routine application [12,13,14,15]. These limitations highlight the need for rapid molecular diagnostic strategies that combine high analytical performance with operational simplicity and the potential for decentralized testing. Recombinase-mediated isothermal amplification (MIRA) is an isothermal nucleic acid amplification technology that enables rapid target amplification at a constant temperature, offering advantages in reaction speed, operational simplicity, and compatibility with portable diagnostic formats [16]. MIRA-based assays have been successfully developed for the detection of various veterinary pathogens [17,18,19], demonstrating their potential for rapid and portable molecular diagnostics. However, amplification alone does not inherently provide sequence-specific signal generation, and the integration of an additional highly specific recognition module could further improve the analytical specificity and robustness of MIRA-based detection.

CRISPR-associated (Cas) proteins have recently emerged as powerful molecular diagnostic tools owing to their programmable nucleic acid recognition and, for certain Cas effectors, collateral cleavage activities [20,21,22]. In particular, Cas12-family nucleases can recognize specific double-stranded DNA targets through guide RNA-directed sequence recognition and, upon activation, exhibit nonspecific trans-cleavage of single-stranded DNA reporters, enabling fluorescence-based signal generation. Integration of CRISPR/Cas-mediated detection with isothermal amplification has led to several highly sensitive diagnostic platforms, including SHERLOCK [23], DETECTR [24] and HOLMES [25]. However, many amplification–CRISPR workflows rely on sequential or physically separated amplification and detection steps, which may require additional pipetting and tube opening and consequently increase operational complexity and the risk of amplicon contamination. Thus, integrating rapid isothermal amplification and CRISPR-mediated detection within a closed, one-pot format represents an attractive strategy for developing rapid and contamination-minimized molecular biosensors.

To address these limitations, we developed a one-pot MIRA-CRISPR/Cas12b fluorescence biosensing platform for rapid detection of *B. canis*, targeting the conserved *omp31* gene. The platform integrates MIRA-mediated isothermal amplification with Cas12b-mediated programmable target recognition and collateral cleavage in a closed reaction system, enabling fluorescence and visual detection while reducing operational steps. This closed reaction format provides a theoretical advantage in minimizing aerosol-mediated amplicon contamination by avoiding post-amplification tube opening [26,27]. The optimized assay achieved a detection limit of 5 copies/reaction within 20 min and demonstrated excellent analytical specificity and clinical agreement with qPCR. With its rapid reaction time, one-pot configuration, and endpoint visual fluorescence readout, the platform provides a simplified molecular diagnostic approach for *B. canis* detection and a basis for further development toward decentralized veterinary testing and One Health surveillance.

## 2. Materials and Methods

### 2.1. Bacterial and Virus Strains, and Clinical Samples

*B. canis* nucleic acid was obtained from the Institute of Animal Sciences, Chinese Academy of Agricultural Sciences. Canine parvovirus (CPV), canine distemper virus (CDV), canine adenovirus (CAV), canine parainfluenza virus (CPIV), *Bordetella bronchiseptica*, *Mycoplasma canis*, and *canine Escherichia coli* strains were maintained and preserved in the laboratory at the Institute of Animal Husbandry and Veterinary Sciences, Zhejiang Academy of Agricultural Sciences. Nucleic acids were extracted from purified viral preparations using an automated nucleic acid extraction system and stored at −80 °C until further use.

For recombinant plasmid construction, the full-length *omp31* gene fragment was cloned into the pMD-19T vector using the Ultra-Universal TOPO Cloning Kit (Vazyme Biotech Co., Ltd., Nanjing, China), generating the recombinant plasmid pMD-*omp31*. The recombinant plasmid was purified, quantified, and stored at −20 °C until use.

A total of 128 clinical canine blood samples were collected from pet hospitals in Zhejiang Province and used for clinical validation of the developed biosensor.

### 2.2. Design and Screening of MIRA Primers and sgRNAs

The *omp31* gene, which encodes a conserved outer membrane protein of *B. canis*, was selected as the target region for specific molecular recognition. Three sgRNAs targeting the *omp31* gene of *B. canis* were designed and synthesized by SynsorBio (Beijing, China) (Table S1). Final sgRNAs were selected based on experimental validation. Using the DNA of *B. canis* as the template, 1 µL of sgRNA (100 ng/µL) was incorporated into the reaction. Following the manufacturer’s instructions (Anpu Future, Changzhou, China). The reaction composition and operating conditions are summarized in Table S2. Briefly, *B. canis* DNA was used as the target template, and FAM fluorescence was monitored at 1 min intervals using an ABI QuantStudio 5 Real-Time PCR System (Thermo Fisher Scientific, Waltham, MA, USA).

MIRA primers targeting different regions of *omp31* were designed using Primer Premier 5.0 software (PREMIER Biosoft International, Palo Alto, CA, USA) (Table S1). The primer pairs were screened based on amplification efficiency by MIRA followed by agarose gel electrophoresis. The MIRA reaction was prepared according to the manufacturer’s instructions, and the reaction composition and amplification conditions are summarized in Table S3.

### 2.3. Optimization of the MIRA-to-CRISPR/Cas12b Reaction Ratio

To determine the optimal proportion of MIRA amplification and CRISPR/Cas12b detection components, *B. canis* DNA was used as the template with the selected MIRA primer pair. Different volumes of the MIRA reaction mixture (5, 10, 15, and 20 μL) were combined with a fixed volume of the CRISPR/Cas12b reaction system (10 μL), and all components were incubated simultaneously in the same reaction tube. The optimal MIRA-to-CRISPR/Cas12b ratio was determined based on the fluorescence signal intensity and signal-to-background discrimination. Based on the optimized ratio, the MIRA amplification and CRISPR/Cas12b detection components were integrated into a single closed tube reaction for the final biosensing assay.

### 2.4. Optimization of Reaction Temperature

To determine the optimal operating reaction temperature of the integrated MIRA-CRISPR/Cas12b biosensing system, *B. canis* DNA templates at high and low concentrations were tested at temperatures ranging from 25 to 50 °C in 5 °C increments. Fluorescence signals were monitored under each temperature condition, and the optimal temperature was determined based on fluorescence intensity and discrimination between positive and negative reactions.

### 2.5. Sensitivity and Specificity Evaluation

The recombinant plasmid pMD-*omp31* was quantified using an ND-2000c spectrophotometer (NanoDrop, Madison, MI, USA), and the plasmid concentration was converted to copy number based on the molecular weight of the recombinant plasmid. Serial dilutions were prepared at concentrations ranging from 5 × 10^4^ to 5 × 10^−2^ copies/reaction for analytical sensitivity evaluation of the one-pot MIRA-CRISPR/Cas12b fluorescence biosensing platform. Sterile nuclease-free water was used as the negative control (NC). Fluorescence signals were monitored using a real-time PCR instrument, and the minimal detectable template copy number that produced a distinguishable fluorescence signal from the NTC under the optimized reaction conditions.

To evaluate analytical specificity, nucleic acids from CPV, CDV, CAV, CPIV, *B. bronchiseptica*, *M. canis*, and canine-origin *E. coli* were tested using the optimized MIRA-CRISPR/Cas12b biosensing platform. *B. canis* DNA was included as the positive control, and nuclease-free water was used as the NC. Fluorescence signals were analyzed using an ABI QuantStudio 5 Real-Time PCR System (Thermo Fisher Scientific, Waltham, MA, USA) and further confirmed by visual fluorescence observation.

### 2.6. Optimization of Reaction Time

Based on the analytical sensitivity results, high- and low-copy-number *omp31* plasmid templates were selected to optimize the reaction time. of the MIRA-CRISPR/Cas12b assay. Reactions were performed for 10, 15, and 20 min under the optimized conditions. Fluorescence signals were monitored using a real-time PCR instrument, and the corresponding fluorescence results were further evaluated by visual observation. The shortest reaction time that provided clear discrimination between positive and negative reactions was selected as the optimal detection time.

### 2.7. Clinical Sample Validation

Nucleic acids were extracted from 128 clinical canine blood samples and tested in parallel using the qPCR reference method (Table S4) and the one-pot MIRA-CRISPR/Cas12b fluorescence biosensing platform (Table S5). The qPCR results were used as the reference standard for evaluating the diagnostic performance of the developed biosensor. Diagnostic sensitivity, specificity, and overall agreement between the two methods were calculated based on the clinical test results.

### 2.8. Statistical Analysis

Statistical analyses were performed using GraphPad Prism 9.4 (GraphPad Software, Boston, MA, USA). Quantitative data are presented as the mean ± standard deviation (SD) of at least three independent experiments.

## 3. Results and Discussion

### 3.1. Design and Principle of the One-Pot MIRA-CRISPR/Cas12b Biosensing Platform

**Figure 1:**
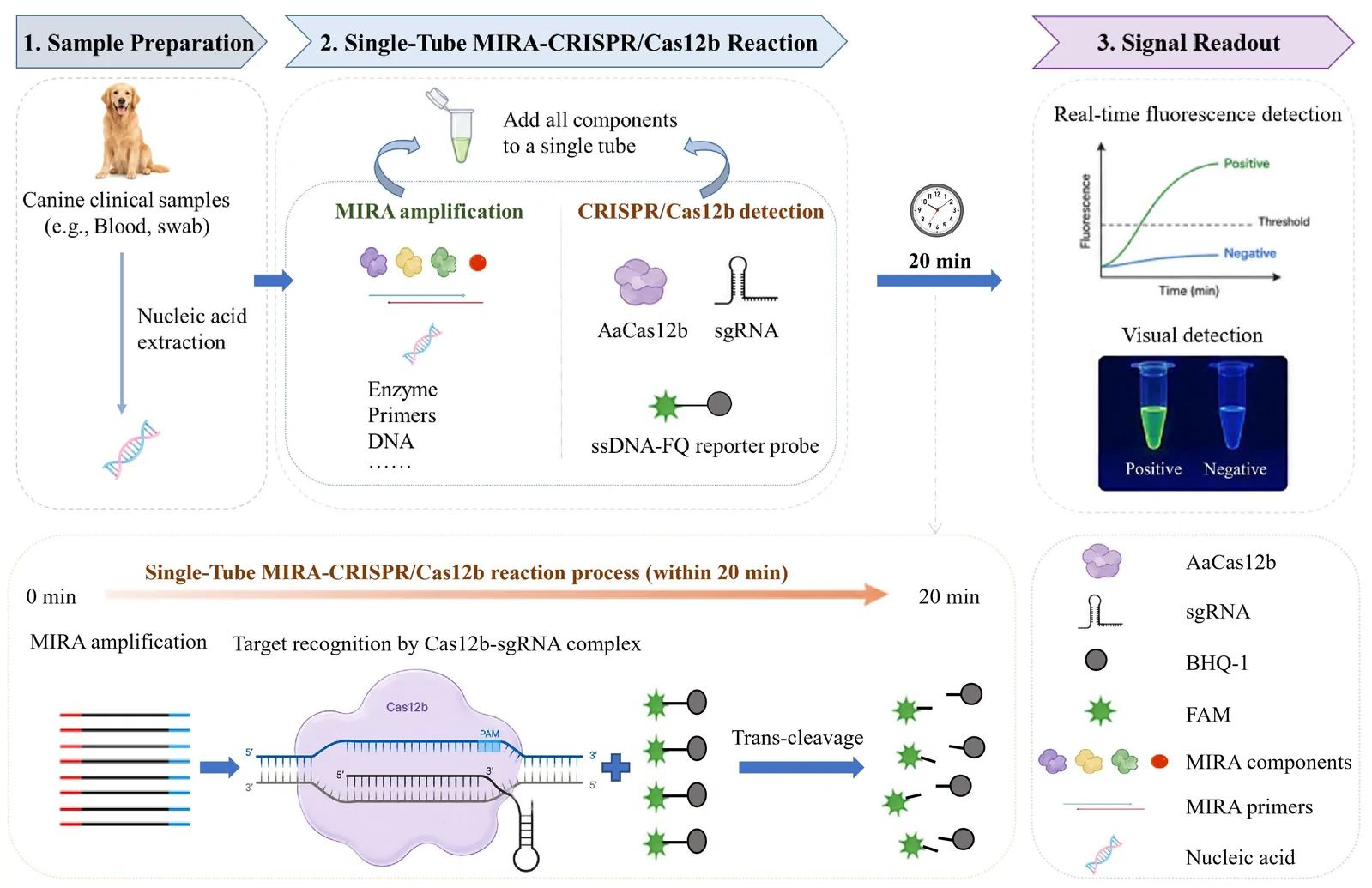
Schematic illustration of the one-pot MIRA-CRISPR/Cas12b fluorescence biosensing platform for rapid detection of *B. canis*.

A one-pot MIRA-CRISPR/Cas12b biosensing platform was developed for the rapid detection *B. canis* (Figure 1). In the assay configuration, all components required for MIRA-mediated amplification and CRISPR/Cas12b-based detection were combined in the same reaction tube before incubation, allowing the two reaction processes to proceed within a single closed reaction system without intermediate tube opening, liquid transfer, or product handling. Following target amplification, the Cas12b-sgRNA complex specifically recognizes the amplified target and activates collateral cleavage of the ssDNA-FAM reporter, resulting in a measurable fluorescence signal. The assay integrates MIRA-mediated isothermal amplification with CRISPR/Cas12b-based target recognition and fluorescent signal generation in a single reaction vessel. The integrated design enables target amplification, sequence specific recognition, and signal generation without opening the reaction tube, thereby simplifying the detection workflow. By eliminating intermediate tube opening and product transfer, this closed tube configuration provides a theoretical advantage in minimizing opportunities for aerosol mediated cross-contamination. The assay was subsequently optimized to achieve rapid fluorescence-based and visual detection of *B. canis.*

### 3.2. Optimization of Recognition and Amplification Components for the One-Pot MIRA-CRISPR/Cas12b Biosensor

**Figure 2:**
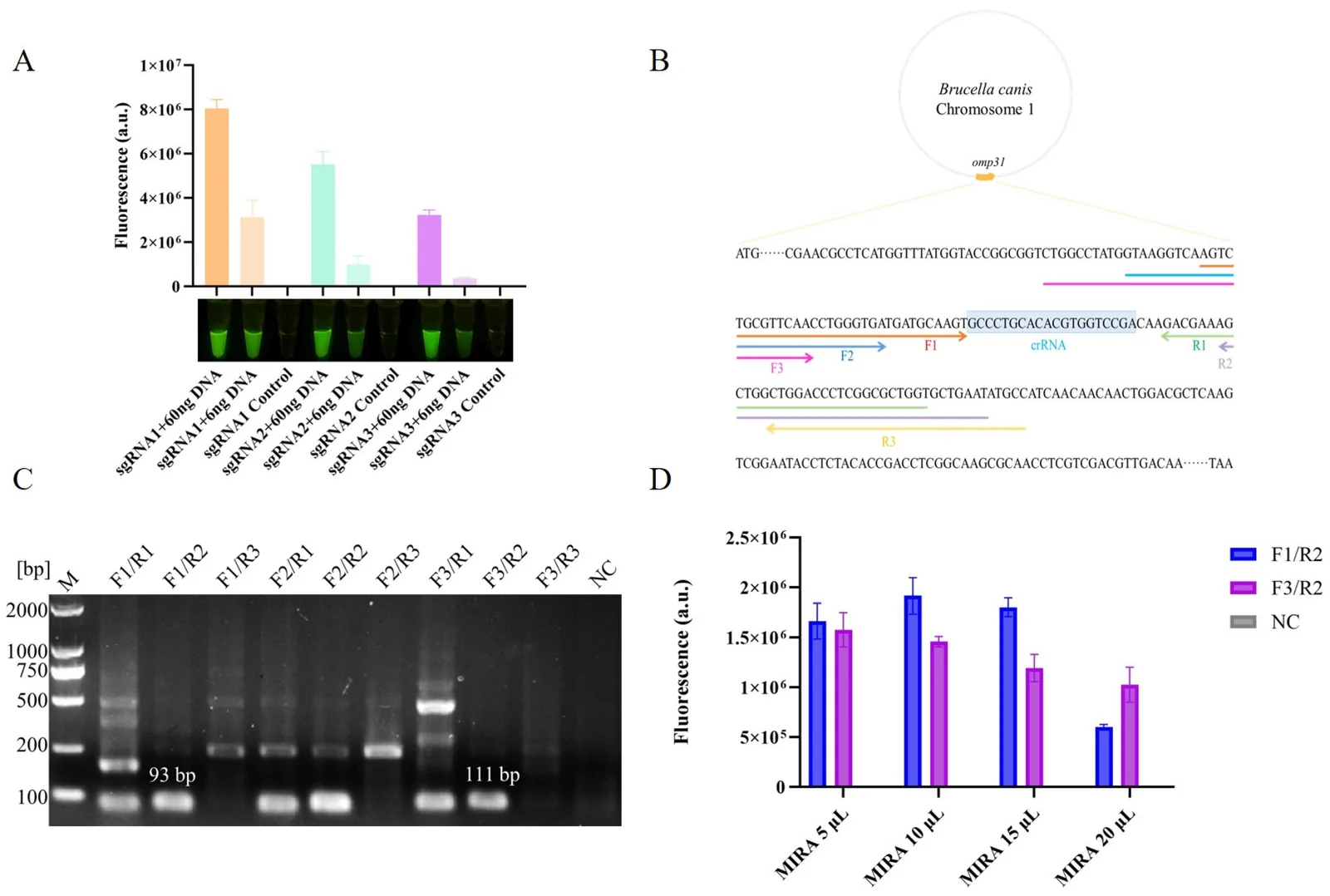
Optimization of target recognition and isothermal amplification parameters of the one-pot MIRA-CRISPR/Cas12b biosensing platform. (**A**) Screening of sgRNAs for the CRISPR/Cas12b-mediated recognition of the *B. canis omp31* target. (**B**) Design of MIRA primers and CRISPR/Cas12b sgRNAs targeting the *omp31* gene. A partial sequence of the *omp31* gene (435–659 bp) is shown. The spacer region of crRNA1 is highlighted in blue. Rightward and leftward arrows indicate primers targeting the sense and antisense strands, respectively. (**C**) Agarose gel electrophoresis analysis of amplification products generated using nine pairs of MIRA primers. M, DNA marker. (**D**) Optimization of the MIRA-to-CRISPR/Cas12b concentration ratio. Error bars represent the standard deviation (SD) from three independent experiments.

The conserved *omp31* gene was selected as the target for *B. canis* detection. Since sgRNA design directly affects Cas12b-mediated sensing efficiency, three sgRNAs were evaluated using different amounts of genomic DNA (60 and 6 ng). Although all sgRNAs activated Cas12b collateral cleavage, sgRNA1 generated the strongest and most consistent fluorescence signals (Figure 2A), indicating its superior target recognition efficiency and suitability for subsequent assay development.

To optimize the MIRA amplification module, nine primer pairs targeting the *omp31* region recognized by sgRNA1 were designed and evaluated. The corresponding primer-binding regions are shown in Figure 2B. Agarose gel electrophoresis showed that only the F1/R2 and F3/R2 primer pairs generated distinct amplification products (Figure 2C). These two primer pairs were subsequently evaluated in the integrated MIRA-CRISPR/Cas12b system. Although both primer pairs supported target amplification, F1/R2 generated a substantially stronger fluorescence response than F3/R2 (Figure 2D), indicating more efficient coupling between MIRA amplification and downstream Cas12b-mediated signal generation. Thus, F1/R2 was selected as the optimal primer pair for subsequent experiments.

The relative contribution of the MIRA amplification and CRISPR/Cas12b detection modules was further optimized by varying the volume of the MIRA amplification reaction while maintaining a constant volume of the CRISPR/Cas12b detection mixture. Among the tested configurations, a 1:1 volume ratio containing 10 μL of MIRA amplification mixture and 10 μL of CRISPR/Cas12b detection mixture produced the highest fluorescence signal (Figure 2D). This result indicates that the relative abundance of amplified target and Cas12b detection components is an important determinant of signal output in the integrated system. Accordingly, sgRNA1, the F1/R2 primer pair, and the optimized MIRA-to-CRISPR/Cas12b reaction configuration (Table S5) were selected for subsequent analytical evaluation.

It should be noted that the one-pot integration of isothermal amplification and CRISPR/Cas systems inevitably introduces potential interference between the nuclease activity of the Cas protein and the amplification reaction. Similar concerns have been reported in one-pot RPA-CRISPR/Cas12a assays, in which the cis-cleavage activity of Cas12a was considered a key factor limiting detection sensitivity and was alleviated by lowering the Cas12a concentration and optimizing the reaction temperature. In our MIRA-CRISPR/Cas12b system, we consider that the cleavage of double-stranded MIRA amplicons by Cas12b is a crRNA-guided, PAM-dependent cis process, whereas the trans activity of activated Cas12b predominantly cleaves ssDNA reporters rather than non-specifically digesting amplification templates. Nevertheless, kinetic competition among MIRA amplicon accumulation, Cas12b activation, and reporter consumption may still occur. To address this, we systematically optimized the mixing ratio of the MIRA and CRISPR/Cas12b systems and the concentrations of Cas12b, crRNA, and reporter, thereby establishing a kinetic window in which MIRA amplification precedes and supports subsequent Cas12b-mediated signal generation. However, strategies such as physical separation of amplification and detection, photocontrolled activation of crRNA or Cas protein, or the addition of glycerol were not applied in this study [28]; these approaches, together with attenuated Cas12b variants, may further improve the performance of one-pot MIRA–CRISPR assays and will be explored in future work.

### 3.3. Optimization of Reaction Temperature for the One-Pot MIRA-CRISPR/Cas12b Biosensor

**Figure 3:**
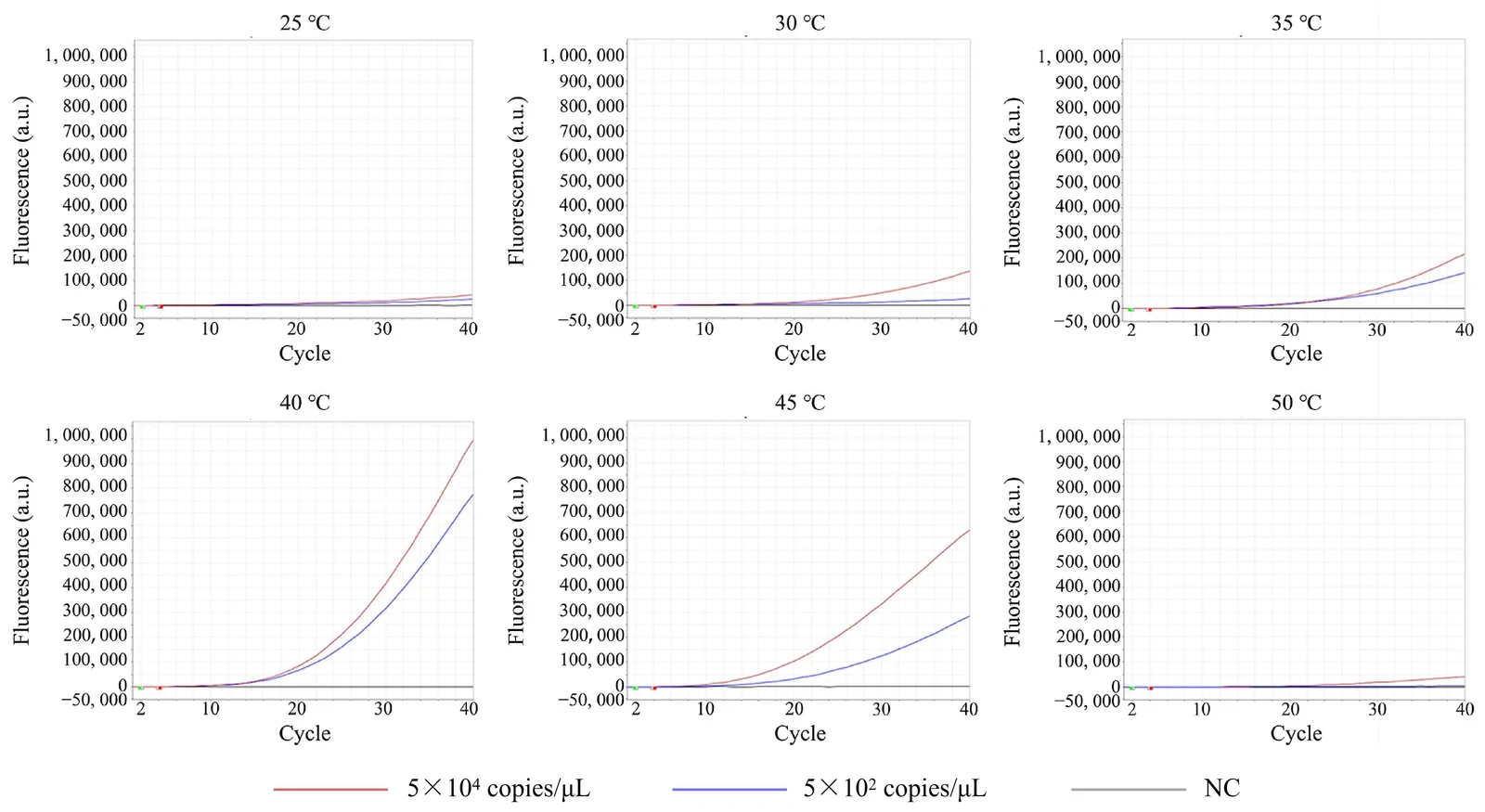
Optimization of the reaction temperature for the MIRA-CRISPR/Cas12b fluorescence biosensing platform. The assay was performed using *B. canis* target DNA at concentrations of 5 × 10^4^ copies/reaction (red lines) and 5 × 10^2^ copies/reaction (blue lines), with a non-template control (gray lines). “Cycle” represents sequential fluorescence acquisition points generated by the instrument software. Fluorescence was recorded every 30 s (2 cycles/min); thus, 40 cycles correspond to 20 min of reaction time. Green and red squares indicate the start and end cycles, respectively, used for baseline determination in the QuantStudio software 1.6.0.

The reaction temperature is a critical parameter governing the compatibility of MIRA amplification with Cas12b-mediated signal generation in the integrated system. Therefore, assay performance was evaluated at temperatures ranging from 25 to 50 °C at 5 °C intervals using two concentrations of *omp31* plasmid templates (5 × 10^4^ and 5 × 10^2^ copies/reaction). The fluorescence output showed a temperature-dependent pattern, with the strongest signals obtained at 40 °C for both template concentrations (Figure 3). Lower temperatures resulted in insufficient signal generation, likely due to reduced amplification efficiency, whereas higher temperatures may negatively affect the activity of the CRISPR/Cas12b sensing module. The enhanced fluorescence output at 40 °C likely resulted from the optimal balance between MIRA-mediated target amplification and Cas12b collateral cleavage activity. Accordingly, 40 °C was selected as the optimal operating temperature for subsequent experiments.

### 3.4. Analytical Sensitivity and Specificity of the One-Pot MIRA-CRISPR/Cas12b Biosensor

**Figure 4:**
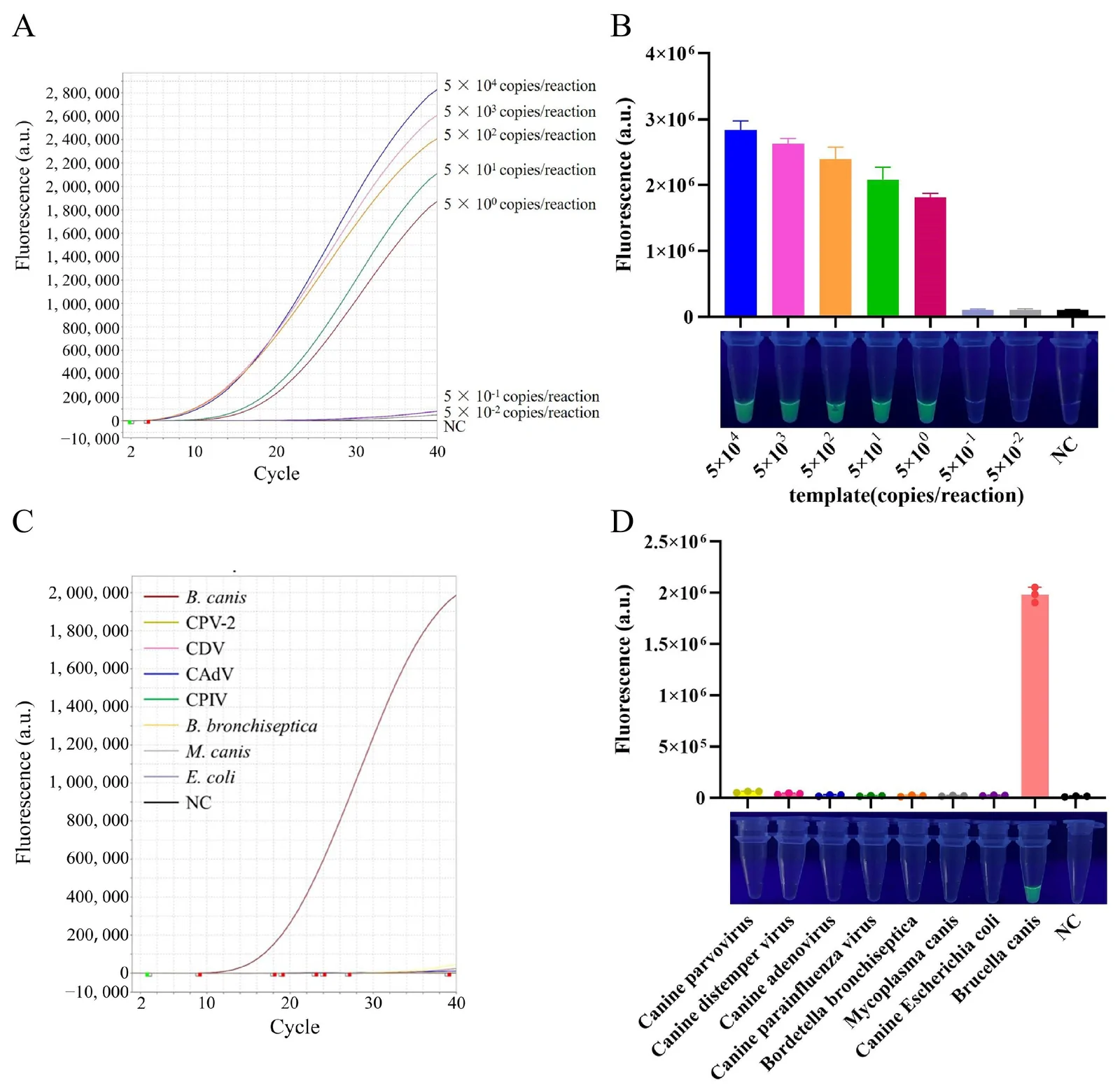
Analytical performance evaluation of the one-pot MIRA-CRISPR/Cas12b fluorescence biosensing platform for *B. canis* detection. (**A**) Real-time fluorescence curves of the MIRA-CRISPR/Cas12b assay using serially diluted recombinant plasmids containing the *omp31* gene. Template concentrations ranging from 5 × 10^4^ to 5 × 10^−2^ copies/reaction. “Cycle” represents sequential fluorescence acquisition points generated by the instrument software. Fluorescence was recorded every 30 s (2 cycles/min); thus, 40 cycles correspond to 20 min of reaction time. The same definition applies to the “Cycle” label in (**C**). Green and red squares in panels (**A**,**C**) indicate the start and end cycles, respectively, used for baseline determination in the QuantStudio software 1.6.0. (**B**) Endpoint fluorescence intensity and visual fluorescence readout of the assay with different concentrations of recombinant plasmids. The detection limit was determined based on the lowest concentration producing a distinguishable fluorescence signal compared with the negative control. (**C**) Specificity evaluation of the MIRA-CRISPR/Cas12b biosensor using genomic DNA from *B. canis* and other common canine pathogens, including canine parvovirus, canine distemper virus, canine adenovirus, canine parainfluenza virus, *Bordetella bronchiseptica*, *Mycoplasma canis*, and *Escherichia coli*. (**D**) Endpoint fluorescence analysis and visual detection results demonstrating the specificity of the biosensor. Error bars represent the standard deviation (SD) from three independent experiments.

The analytical sensitivity of the one-pot MIRA-CRISPR/Cas12b biosensor was evaluated using serially diluted *omp31* plasmid templates under the optimized conditions. A clear fluorescence signal was obtained within 20 min at a concentration of 5 copies per reaction, which was further confirmed by visual fluorescence detection under blue light illumination (Figure 4A,B). To benchmark the analytical sensitivity of the developed biosensor against a conventional molecular diagnostic method, the same serially diluted plasmid templates were analyzed in parallel by qPCR. It should be noted that the 5 copies/reaction reported here represents the minimal detectable template copy number based on three independent serial dilution tests, rather than the probabilistic LOD evaluated by probit regression with ≥95% detection probability following CLSI EP17 guidelines. The qPCR assay also exhibited a detection limit of 5 copies/reaction (Figure S1), indicating that the analytical sensitivity of the one-pot MIRA-CRISPR/Cas12b biosensor was comparable to that of qPCR under the conditions evaluated. Although the biosensor did not provide a lower detection limit than qPCR, its ability to achieve equivalent analytical sensitivity within a simplified one-pot isothermal workflow highlights its potential as a rapid alternative for *B. canis* detection. The combination of MIRA-mediated target amplification and Cas12b-triggered collateral cleavage enables efficient conversion of low-copy-number nucleic acid targets into readily detectable fluorescence signals.

The analytical specificity was assessed using nucleic acids from seven common canine pathogens, including canine parvovirus, canine distemper virus, canine adenovirus, canine parainfluenza virus, *Bordetella bronchiseptica*, *Mycoplasma canis*, and *Escherichia coli*. Only *B. canis*-positive samples generated detectable fluorescence signals, while all non-target pathogens remained negative (Figure 4C,D). No detectable cross-reactivity was observed with the tested canine pathogens, supporting the suitability of the biosensor for specific detection of *B. canis* in samples containing potentially interfering nucleic acids.

Because canine brucellosis is primarily associated with *B. canis*, the present study focused experimentally on the detection of *B. canis*. Nevertheless, previous studies have documented occasional cross-species spillover of other Brucella species into dogs, including *B. melitensis* and *B.suis*, although such infections appear to occur at a substantially lower frequency [29,30]. Epidemiological evidence has also indicated transmission involving the sheep–dog–human interface, highlighting the potential role of dogs in the circulation of Brucella between animal reservoirs and humans [31]. These findings underscore the epidemiological relevance of considering other Brucella species when interpreting molecular detection results in dogs. To further assess the potential for cross-reactivity with closely related Brucella species, an in silico sequence alignment of the *omp31* target region, including the MIRA primer-binding and CRISPR/Cas12b recognition regions, was performed. The analysis showed relatively high sequence conservation among *B. canis*, *B. suis*, *B. melitensis*, *B. ceti*, and *B. ovis* (Figure S2). The high conservation of the targeted *omp31* region suggests that, in addition to *B. canis*, other closely related *Brucella* species carrying homologous sequences may also be detected by the developed biosensor. Although such cross-species infections in dogs are relatively uncommon, this potential cross-reactivity may allow the assay to detect occasional infections caused by other closely related *Brucella* species. As *omp31* is a relatively conserved gene in *B. canis*, we further evaluated the conservation of the regions targeted by the MIRA primers and CRISPR/Cas12b among *B. canis* strains from different temporal and geographic origins. Both target regions showed 100% sequence identity among the strains examined (Figure S3), supporting the conservation of the selected target region for *B. canis* detection.

Collectively, the one-pot MIRA-CRISPR/Cas12b biosensor achieved analytical sensitivity comparable to qPCR while integrating target amplification, sequence-specific recognition, and fluorescence signal generation into a single reaction. Although a complete field-deployable workflow would require integration with appropriate portable sample-preparation and detection technologies, together with its rapid detection time and simplified workflow, these characteristics support its potential as an accessible molecular diagnostic tool for *B. canis* detection, particularly in point-of-care and field-based settings.

### 3.5. Optimization of Detection Time for the One-Pot MIRA-CRISPR/Cas12b Biosensor

**Figure 5:**
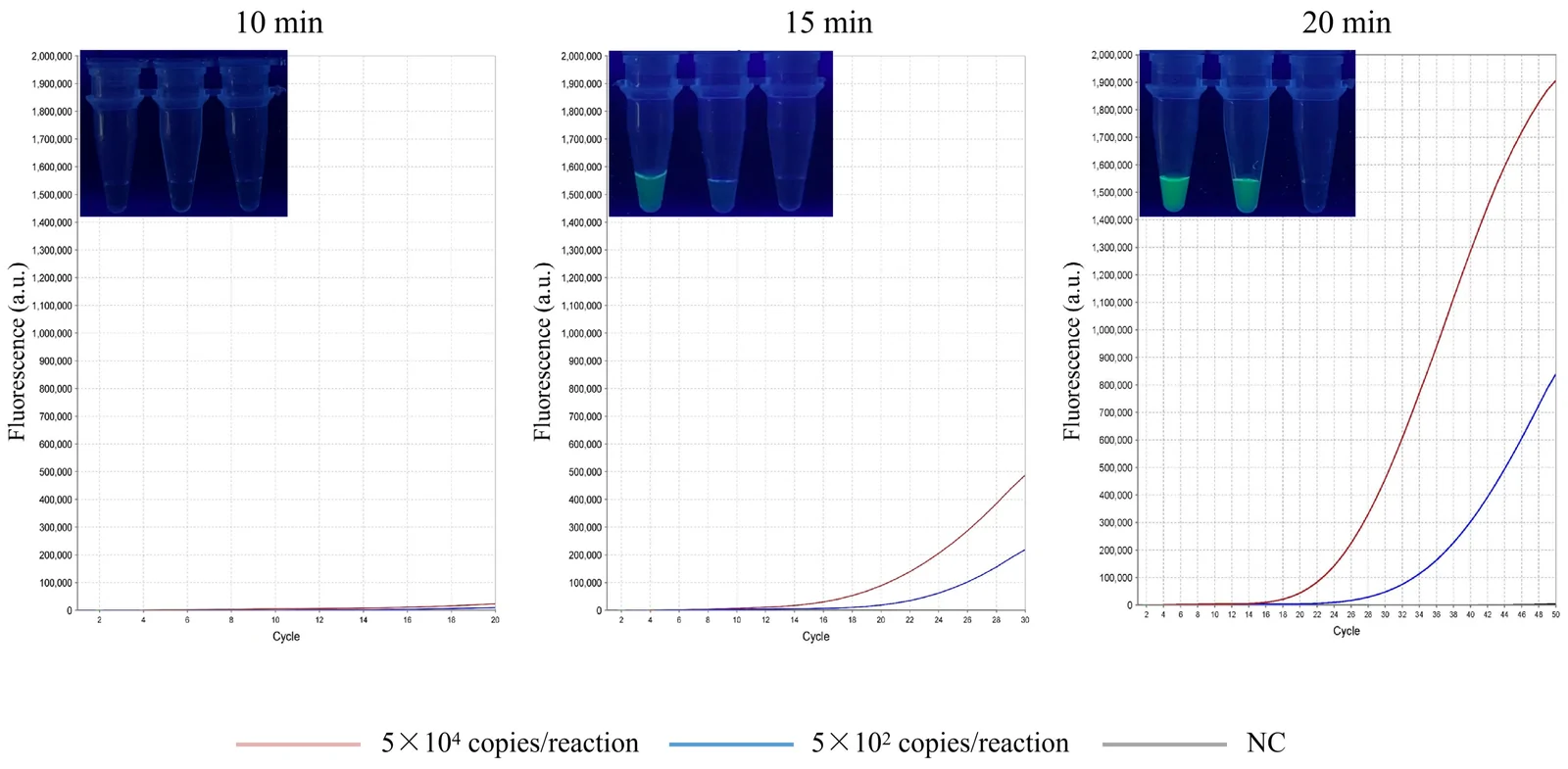
Optimization of the reaction time for the one-pot MIRA-CRISPR/Cas12b fluorescence biosensing platform. The assay was performed using *B. canis* target DNA at concentrations of 5 × 10^4^ copies/reaction (red lines) and 5 × 10^2^ copies/reaction (blue lines), with a non-template control (gray lines). “Cycle” represents sequential fluorescence acquisition points generated by the instrument software. Fluorescence was recorded every 30 s (2 cycles/min); thus, 40 cycles correspond to 20 min of reaction time.

To determine the minimum reaction time required for reliable target detection, high- and low-copy-number *omp31* plasmid templates (5 × 10^4^ and 5 × 10^2^ copies/reaction) were selected based on the analytical sensitivity results and evaluated at reaction times of 10, 15, and 20 min. After 10 min, fluorescence signals at both template concentrations remained close to the negative control, suggesting insufficient target amplification and signal accumulation. At 15 min, a distinct fluorescence response was observed for the higher template concentration, whereas the lower concentration remained weakly differentiated from the background. Extending the reaction to 20 min produced pronounced and clearly distinguishable fluorescence signals for both concentrations, which were also readily visualized under blue light illumination (Figure 5). These results demonstrate that a 20 min reaction provides sufficient time for coordinated MIRA-mediated target amplification and Cas12b-mediated signal generation while maintaining a rapid assay workflow. Accordingly, 20 min was selected as the optimal detection time for the one-pot MIRA-CRISPR/Cas12b biosensor.

### 3.6. Clinical Validation of the One-Pot MIRA-CRISPR/Cas12b Biosensor

**Figure 6:**
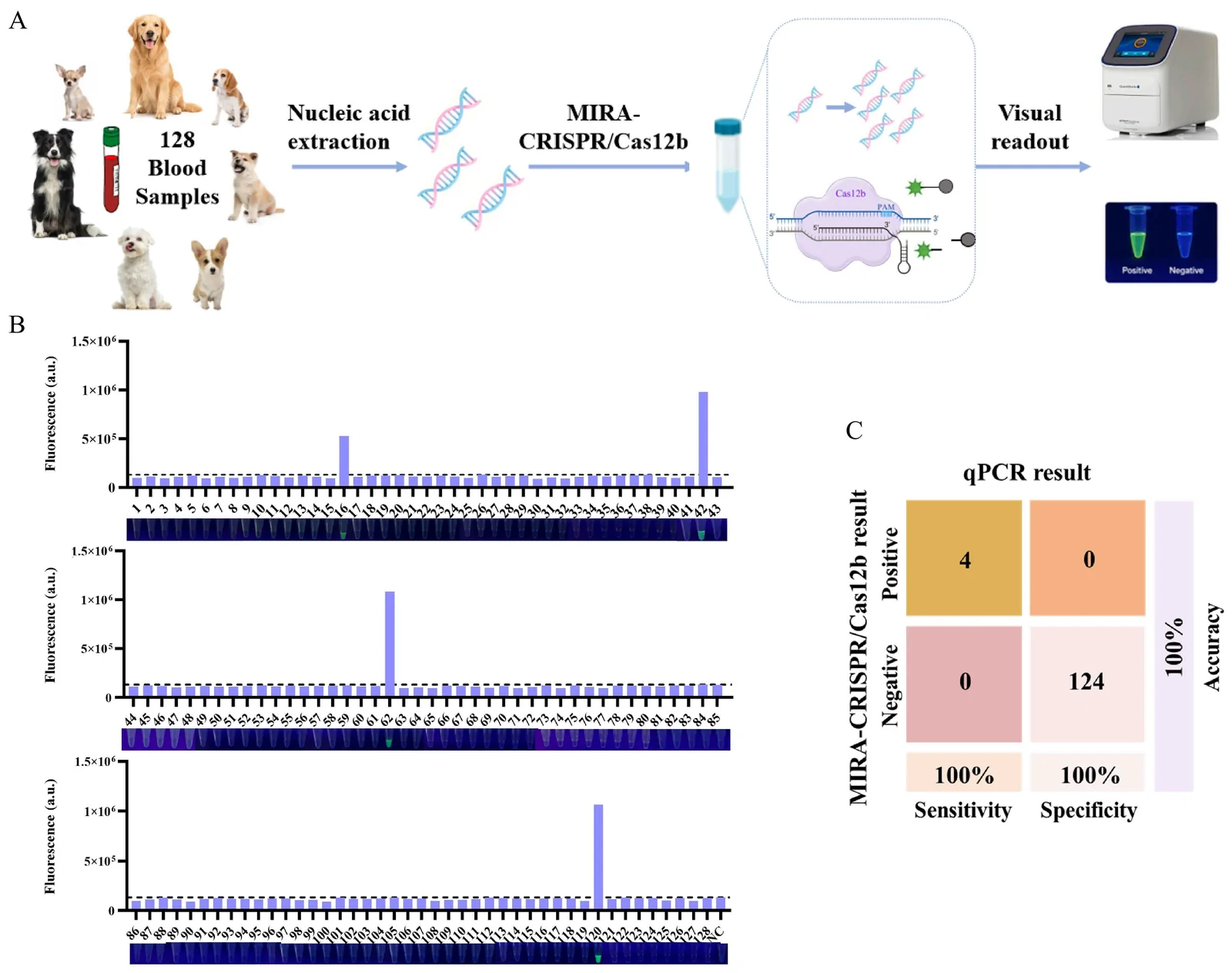
Clinical validation of the one-pot MIRA-CRISPR/Cas12b fluorescence biosensing platform for *B. canis* detection. (**A**) Schematic workflow of clinical sample detection using the one-pot MIRA-CRISPR/Cas12b biosensing platform. Genomic DNA extracted from canine blood samples was directly subjected to MIRA-mediated amplification and CRISPR/Cas12b-based fluorescence detection, followed by visual fluorescence readout. (**B**) Detection results of 128 clinical blood samples analyzed using the MIRA-CRISPR/Cas12b biosensor. Fluorescence intensity and corresponding visual readouts were used to determine positive and negative samples. The dashed line indicates the threshold for positive signal interpretation. (**C**) Comparison of the MIRA-CRISPR/Cas12b assay with qPCR for clinical sample validation.

To evaluate the practical applicability of the developed biosensor, 128 clinical blood samples were analyzed in parallel using the one-pot MIRA-CRISPR/Cas12b biosensor and qPCR as the reference method (Figure 6A). Nucleic acids extracted from the clinical specimens were directly subjected to both assays, and fluorescence signals were evaluated relative to the negative control background (Figure 6B). Among the 128 clinical samples, the biosensor identified 4 positive and 124 negative samples, showing complete agreement with qPCR results. The diagnostic sensitivity was 100% (95% CI: 39.8–100%), while the diagnostic specificity was 100% (95% CI: 97.1–100%) relative to the reference method (Figure 6C).

In addition to instrument-based fluorescence monitoring, endpoint fluorescence was also evaluated under blue light illumination. A clearly visible fluorescence signal was observed at the analytical detection limit of 5 copies/reaction, and the endpoint visual fluorescence results of the clinical samples were consistent with the corresponding fluorescence measurements obtained using the real-time instrument. These findings support the feasibility of using endpoint visual fluorescence as a simpler alternative to instrument-based fluorescence monitoring. These results demonstrate that the biosensor maintains reliable analytical performance in clinical specimens, indicating its potential for practical *B. canis* detection. While real-time fluorescence monitoring requires laboratory-grade instrumentation, the endpoint visual readout under blue light illumination may facilitate decentralized veterinary testing. Portable and simplified nucleic acid extraction technologies have been increasingly developed for point-of-care molecular diagnostics. However, the compatibility of such sample-preparation approaches with the present MIRA-CRISPR/Cas12b platform for canine blood specimens remains to be evaluated. Further evaluation combining portable sample-preparation technologies with portable or simplified detection devices, together with larger and geographically diverse clinical cohorts, will be important to assess the feasibility and robustness of the platform for decentralized veterinary applications.

Because whole blood is a complex biological matrix containing potential amplification inhibitors, residual matrix associated inhibitors in extracted nucleic acid preparations may potentially affect downstream amplification. In the present study, nucleic acids were extracted from the clinical blood specimens prior to amplification, and the biosensor results showed complete agreement with those obtained by qPCR, suggesting that no obvious matrix-associated inhibition affected assay performance under the conditions evaluated. Nevertheless, potential sample-dependent effects arising from residual matrix components should be taken into consideration when interpreting results from complex clinical specimens. Further validation using a broader range of clinical samples and appropriate internal controls would help to further assess the robustness of the assay across different sample matrices.

## 4. Conclusions

Canine brucellosis caused by *Brucella canis* has emerged as an important challenge in companion animal health and zoonotic disease surveillance, particularly with the increasing integration of companion animals into human households and the rapid expansion of the pet economy. The often asymptomatic or chronic nature of *B. canis* infection and its zoonotic potential highlight the need for rapid, sensitive, and accessible diagnostic methods. In this study, we developed a one-pot MIRA-CRISPR/Cas12b biosensing platform for rapid detection of *B. canis* by integrating MIRA-mediated isothermal amplification with CRISPR/Cas12b-based target recognition and collateral cleavage. The optimized assay achieved a detection limit of 5 copies/reaction within 20 min, with both real-time fluorescence and visual fluorescence readouts. This configuration distinguishes the present platform from one-pot two-step approaches that require sequential initiation of the amplification and CRISPR detection reactions [16,17]. In the present single-pot configuration, MIRA amplification and Cas12b-mediated signal generation are integrated within the same reaction mixture from the outset, eliminating the need for tube opening, product transfer, or additional manipulation between amplification and detection. This configuration simplifies the overall workflow, while its closed reaction format provides a theoretical advantage in minimizing the risk of aerosol-mediated cross-contamination.

Compared with representative nucleic acid-based detection methods, including digital PCR [32], isothermal amplification assays [33,34,35], and CRISPR-mediated biosensing strategies (Table 1) [36,37], the developed platform provides a favorable combination of rapid detection, analytical sensitivity, and operational simplicity. Because the reported minimal detectable template copy number in previous studies were determined using different analytical units, assay configurations, and target Brucella species, direct cross study comparison of detection limits should be interpreted with caution. Within the conditions of the present study, the biosensor achieved a detection limit of 5 copies/reaction, comparable to that of qPCR, while requiring only 20 min and avoiding separate amplification and detection procedures. This combination of sensitivity, speed, and simplified operation may be particularly advantageous for veterinary diagnostic applications requiring rapid testing, while the endpoint visual readout may facilitate decentralized testing.

The clinical applicability of the biosensor was further evaluated using 128 clinical blood samples. The assay identified 4 positive and 124 negative samples, showing complete agreement with qPCR and achieving 100% sensitivity and 100% specificity relative to the reference method. These findings demonstrate that the analytical performance of the platform can be maintained in clinical specimens and support its potential for practical *B. canis* detection. Nevertheless, the limited number of positive specimens (*n* = 4) resulted in a wide confidence interval for the estimated sensitivity. Further validation using larger and geographically diverse clinical cohorts would strengthen the assessment of its diagnostic performance.

Collectively, the one-pot MIRA-CRISPR/Cas12b biosensor provides a rapid, sensitive, and specific molecular diagnostic strategy for *B. canis* detection. Its 20 min detection time, analytical sensitivity comparable to qPCR, closed tube reaction format, and compatibility with both fluorescence and visual readouts support its potential for practical veterinary testing. While real-time fluorescence monitoring requires laboratory-grade instrumentation, the clearly visible endpoint fluorescence signal under blue light illumination provides a simpler readout option and supports the potential use of the assay in decentralized veterinary testing. This platform therefore provides a basis for further development of accessible molecular diagnostics for canine brucellosis within a One Health framework.

## Figures and Tables

**Table 1:** Comparison of representative molecular methods for the detection of Brucella species.

Method	Target Organism	Limit of Detection	Detection Time	Reference
Droplet Digital PCR	*B. abortus* (VAC)	2.13 × 10^4^ copies/μL	~90 min	[32]
LAMP-LFIA	*B. abortus*	100 fg/reaction	85 min	[34]
MCDA-LFB	*B. abortus*	3 copies/μL	70 min	[35]
LAMP-LFIA	*B. melitensis*	12.1 fg/reaction	60 min	[33]
RPA-CRISPR/Cas12a	*B. melitensis*	1 copies/μL	~60 min	[37]
CRISPR/Cas12a-MCDA-LFB	*B. melitensis*	2 copies/μL	90 min	[36]
MIRA-CRISPR/Cas12b	*B. canis*	5 copies/reaction (5 copies/μL)	20 min	This work

Note: minimal detectable concentration values are reported according to the original publications. Because the included studies differ in template input volume, assay configuration, target Brucella species, and analytical units, the reported minimal detectable concentration should be interpreted as reported performance characteristics rather than directly comparable sensitivity measures.

## Data Availability

All the data generated during this study are included in the manuscript. Additional data related to this article may be requested from the corresponding authors.

## Supplementary Materials

The following supporting information can be downloaded at: https://www.mdpi.com/article/10.3390/bios16100578/s1, Table S1: Sequences of MIRA primers and sgRNAs used in this study; Table S2: Composition and reaction conditions of the AaCas12b-based fluorescence detection system for sgRNA screening; Table S3: Composition and reaction conditions of the MIRA amplification system; Table S4: Composition and reaction conditions of the qPCR amplification system; Table S5: Composition and reaction conditions of the one-pot MIRA-CRISPR/Cas12b biosensing system; Figure S1: Analytical sensitivity and standard curve of the qPCR assay for *B. canis* detection; Figure S2: Multiple sequence alignment of the *omp31* target region among representative Brucella species; Figure S3: Sequence alignment of the *omp31* target region among *B. canis* strains from different temporal and geographic origins.
