# Supplementary material for: Development of a One-Pot MIRA-CRISPR/Cas12b Fluorescence Biosensing Platform for Rapid Detection of Brucella canis
Source: Biosensors (Basel). 2026 Oct 15;16(10):578. doi: 10.3390/bios16100578 (PMC13702669; doi:10.3390/bios16100578)
Supplement: Supplementary file 1 [file biosensors-16-00578-s001.zip › biosensors-4560528-supplementary.pdf]

biosensors

MDPI

Development of a One-Pot MIRA-CRISPR/Cas12b Fluorescence Biosensing Platform for Rapid Detection of *Brucella canis*

Suxin Huo [1,†], Yinchu Zhu [1,†], Congyun Ji [1], Fu Fan [2], Ruiping Lin [2], Weicheng Ye [1], Yuan Fu [1], Jionggang Hua [1], Gaole Feng [2], Liu Chen [1], Tao Yun [1], Cun Zhang [1], Zhuanli Wu [1], Xing Xu [1], Yue Wu [1,*] and Zheng Ni [1,*]

[1] State Key Laboratory for Quality and Safety of Agro-Products, Key Laboratory of Animal and Poultry Breeding of Zhejiang Province, Key Laboratory of Livestock and Poultry Resources (Poultry) Evaluation and Utilization, Institute of Animal Husbandry and Veterinary Science, Zhejiang Academy of Agricultural Sciences, Hangzhou 310021, China; huosuxin831@163.com (S.H.); zhuyinchu@zaas.ac.cn (Y.Z.)
[2] Pinghu Animal Husbandry and Veterinary Station, Pinghu 314200, China
* Correspondence: wuyue@zaas.ac.cn (Y.W.); niz@zaas.ac.cn (Z.N.)
† These authors contributed equally to this work.

This PDF file include:

Table S1 to S5
Figure S1 and S3

Table S1. Sequences of MIRA primers and sgRNAs used in this study.

Type	Oligonucleotide	Sequence (5' – 3')
sgRNA	sgRNA 1	GUCUAAAGGACAGAUUUUCAACGGGUGUGCCAAUGGCCACU-UUCCAGGUGGCAAA-GCCCGUUGAACUUCAAGCGAAGUGGCACUCGGACCACGUGUGCAGGGC
sgRNA	sgRNA 2	GUCUAAAGGACAGAUUUUCAACGGGUGUGCCAAUGGCCACU-UUCCAGGUGGCAAA-GCCCGUUGAACUUCAAGCGAAGUGGCACCGGGUCCGAUUUCAGCCGG
sgRNA	sgRNA 3	GUCUAAAGGACAGAUUUUCAACGGGUGUGCCAAUGGCCACU-UUCCAGGUGGCAAA-GCCCGUUGAACUUCAAGCGAAGUGGCACCUUGAGAGCAAGGUCAAUUU
MIRA primer	MIRA-F1	AGTCTGCGTTCAACCTGGGTGATGATGCAAGT
MIRA primer	MIRA-F2	GTAAGGTCAAGTCTGCGTTCAACCTGGGTGA
MIRA primer	MIRA-F3	CTGGCCTATGGTAAGGTCAAGTCTGCGTTCAA
MIRA primer	MIRA-R1	ACCAGCGCCGAGGGTCCAGCCAGCTTTCGTC
MIRA primer	MIRA-R2	ATTCAGCACCAGCGCCGAGGGTCCAGCCAGC
MIRA primer	MIRA-R3	GGCATATTCAGCACCAGCGCCGAGGGTCCAG
PCR	omp31-F	AAGTCCGTAATTTTGGCGTCCATC
PCR	omp31-R	AGACCGACGCGAACAGTGTGGAA
qPCR	omp31-qF	GCTGAATATGCCATCAAC
qPCR	omp31-qR	CAAGGAAGCTATTGTCAAC
qPCR	probe	MGB-ACGCTCAAGTCGGAATACCTCTAC-BHQ1

 https://doi.org/10.3390/bios16100578

Table S2. Composition and reaction conditions of the AaCas12b-based fluorescence detection system for sgRNA screening.

Component	Volume (μL)	Final concentration
sgRNA (100 ng/μL)	1	2 ng/μL
AaCas12b (2.5 μM)	2	0.1 μM
10 × AaCas12b Buffer	5	1 ×
CRISPR ssDNA Reporter (12b-FAM) (100 μM)	0.5	1 μM
B. canis DNA	1	-
Nuclease-free water	49.5	-
Total volume	50	-
Reaction conditions	40°C, 15 min	
Detection channel	FAM	

Table S3. Composition and reaction conditions of the MIRA amplification system.

Component	Volume (μL)
Buffer A	29.4
Forward primer (10 μM)	2
Reverse primer (10 μM)	2
B. canis DNA	2
Buffer B	2.5
Nuclease-free water	12.1
Total volume	50
Reaction conditions	40 °C, 20 min

Table S4. Composition and reaction conditions of the qPCR amplification system.

Component	Volume (μL)
2 × Taq Pro HS Universal Probe Master Mix	10
Forward primer (10 μM)	0.4
Reverse primer (10 μM)	0.4
TaqMan Probe (10 μM)	0.2
B. canis DNA	1
Nuclease-free water	8
Total volume	20
Reaction conditions	95 °C, 20 s; 40 cycles: 95 °C, 1 s; 60 °C, 30 s
Interpretation criteria	Positive: Typical amplification curve with Ct ≤35.<br>Negative: No detectable amplification or Ct >35.

Table S5. Composition and reaction conditions of the one-pot MIRA-CRISPR/Cas12b biosensing system.

Reagent	Component	Volume (μL)
MIRA	Buffer A	5.88
	Forward primer (10 μM)	1
	Reverse primer (10 μM)	1
	Buffer B	1
Cas12b	sgRNA(100 ng/μL)	0.2
	AaCas12b (2.5 μM)	0.4
	10 × AaCas12b Buffer	2
	CRISPR ssDNA Reporter (12b-FAM) (100 μM)	0.1
DNA template		1
Nuclease-free water		7.42
Total volume		20
Reaction conditions		40°C, 20 min
Detection channel		FAM

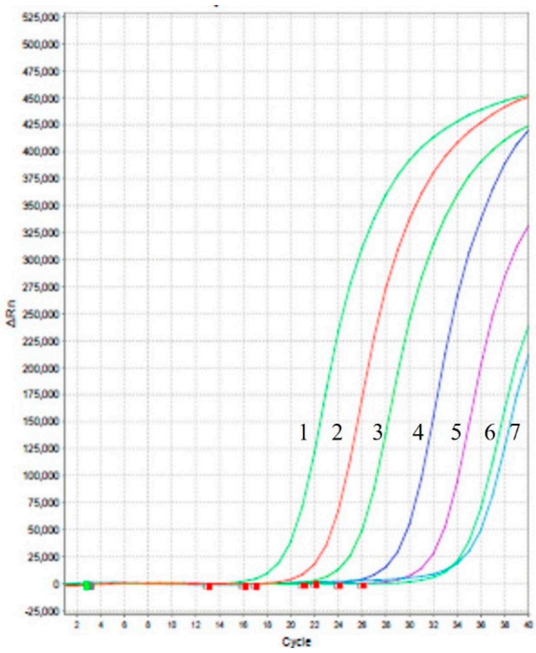

Figure S1. Analytical sensitivity of the qPCR assay for *B. canis* detection. Amplification curves generated from serially diluted *omp31*-containing plasmid templates at concentrations of 5 × 10^4 to 5 × 10^{-2} copies/reaction. Curves 1–7 represent 5 × 10^4, 5 × 10^3, 5 × 10^2, 5 × 10^1, 5 × 10^0, 5 × 10^{-1}, and 5 × 10^{-2} copies/reaction, respectively. Each concentration was tested in triplicate.

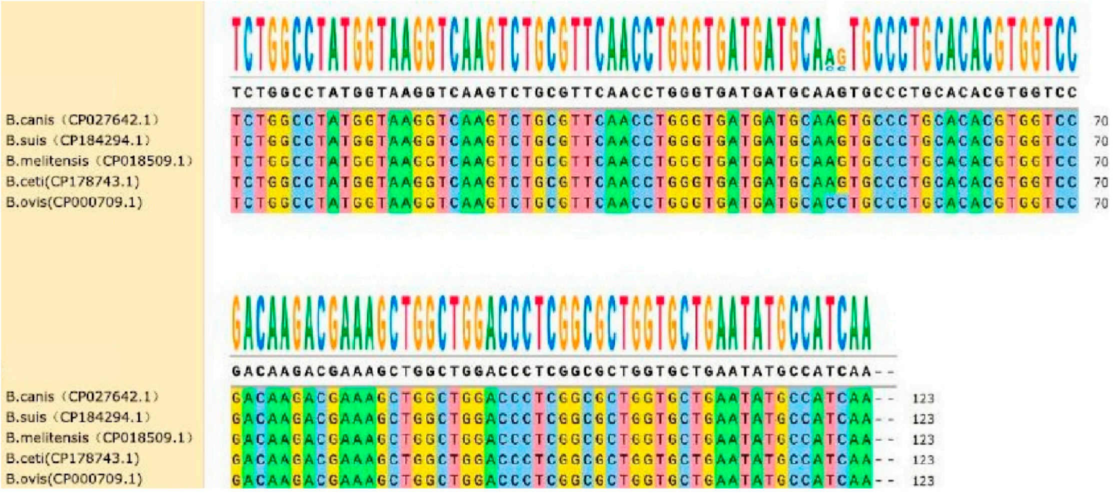

Figure S2. Multiple sequence alignment of the omp31 target region among representative Brucella species. The *omp31* sequences of *B. canis* (CP027642.1), *B. suis* (CP184294.1), *B. melitensis* (CP018509.1), *B. ceti* (CP178743.1), and *B. ovis* (CP000709.1) were aligned using SnapGene 6.0.2 and NCBI BLAST. The regions targeted by the MIRA primers and CRISPR/Cas12b recognition were analyzed for sequence conservation.

https://doi.org/10.3390/bios16100578

Strain name	GenBank ID	Date of isolation	Isolation location
GB1	CP027642.1	2012	China:Beijing
Bc1066	CP184292.1	2024	Kazakhstan: Almaty
2010009751	CP016978.1	2010	USA: Massachusetts
2009004498	CP016974.1	2009	Louisianna
ATCC 23365	CP000873.1	2007	USA
RM6/66	CP007759.1	2014	USA
M-24	CM152614.1	2024	Ukraine: Dnipropetrovsk
A52141	CP003175.1	2011	South Korea: Hwaseong

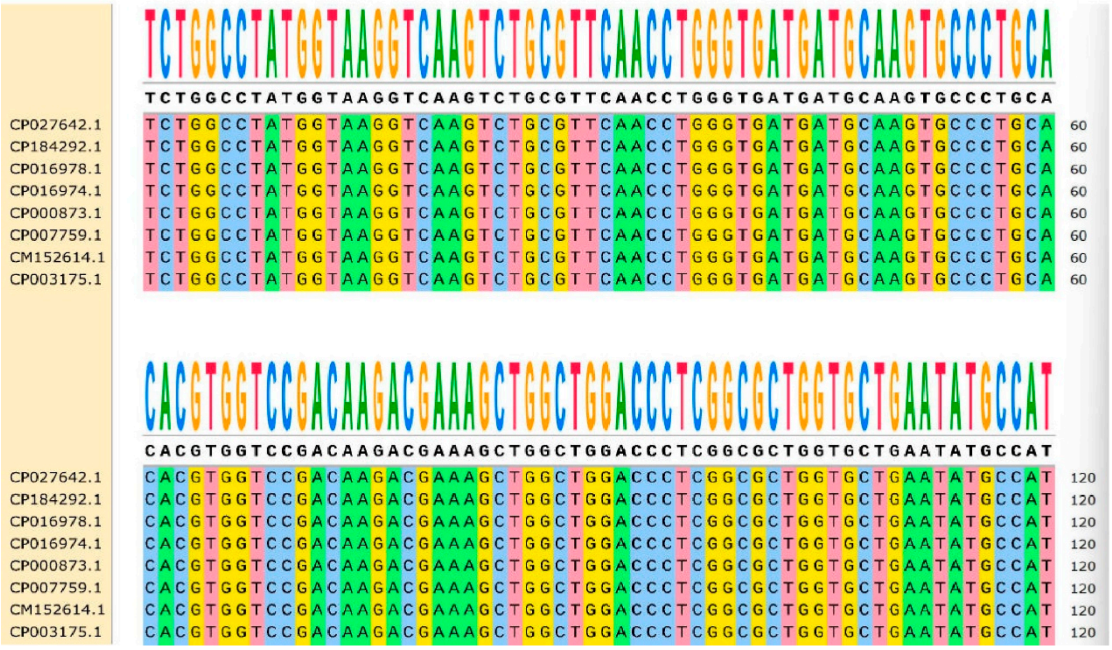

Figure S3. Sequence alignment of the *omp31* target region among *B. canis* strains from different temporal and geographic origins. Representative *omp31* sequences of *B. canis* strains isolated at different times and from different geographic locations were aligned using SnapGene 6.0.2 and NCBI BLAST. The regions targeted by the MIRA primers and CRISPR/Cas12b recognition were analyzed for sequence conservation.

https://doi.org/10.3390/bios16100578
